# Crotoxin B: Heterologous Expression, Protein Folding, Immunogenic Properties, and Irregular Presence in Crotalid Venoms

**DOI:** 10.3390/toxins14060382

**Published:** 2022-05-31

**Authors:** Miguel Angel Mejía-Sánchez, Herlinda Clement, Ligia Luz Corrales-García, Timoteo Olamendi-Portugal, Alejandro Carbajal, Gerardo Corzo

**Affiliations:** 1 Departamento de Medicina Molecular y Bioprocesos, Instituto de Biotecnologia, Universidad Nacional Autónoma de México, Avenida Universidad, 2001, Apartado Postal 510-3, Cuernavaca 62210, Mexico; angel.mejia@ibt.unam.mx (M.A.M.-S.); herlinda.clement@ibt.unam.mx (H.C.); ligialu@gmail.com (L.L.C.-G.); timoteo.olamendi@ibt.unam.mx (T.O.-P.); 2 Departamento de Alimentos, Facultad de Ciencias Farmacéuticas y Alimentarias, Universidad de Antoquia, AA 1226, Medellín 050010, Colombia; 3 Centro de Investigaciones Biológicas del Noroeste, S.C. Instituto Politécnico Nacional s/n, Playa Palo de Santa Rita Sur, La Paz 23096, Mexico; acarbajalsaucedo@gmail.com; 4 Herpetario Kiinam, Colección Científica, Periférico sur 7666-477, Rinconada Coapa, Tlalpan, Ciudad de Mexico 14326, Mexico

**Keywords:** antibodies, *Crotalus tzabcan*, phospholipase, protein expression, snake, venom, viper

## Abstract

Crotoxin complex CA/CB and crotamine are the main toxins associated with *Crotalus* envenomation besides the enzymatic activities of phospholipases (PLA_2_) and proteases. The neutralization at least of the crotoxin complex by neutralizing the subunit B could be a key in the production process of antivenoms against crotalids. Therefore, in this work, a Crotoxin B was recombinantly expressed to evaluate its capacity as an immunogen and its ability to produce neutralizing antibodies against crotalid venoms. A Crotoxin B transcript from *Crotalus tzabcan* was cloned into a pCR^®^2.1-TOPO vector (Invitrogen, Waltham, MA, USA) and subsequently expressed heterologously in bacteria. HisrCrotoxin B was extracted from inclusion bodies and refolded in vitro. The secondary structure of HisrCrotoxin B was comparable to the secondary structure of the native Crotoxin B, and it has PLA_2_ activity as the native Crotoxin B. HisrCrotoxin B was used to immunize rabbits, and the obtained antibodies partially inhibited the activity of PLA_2_ from *C. tzabcan*. The anti-HisrCrotoxin B antibodies neutralized the native Crotoxin B and the whole venoms from *C. tzabcan, C. s. salvini*, and *C. mictlantecuhtli.* Additionally, anti-HisrCrotoxin B antibodies recognized native Crotoxin B from different *Crotalus* species, and they could discriminate venom in species with high or low levels of or absence of Crotoxin B.

## 1. Introduction

Crotoxin is a PLA_2_ isolated and purified originally from the venom of the snake *Crotalus durissus terrificus*. It is generally found as a heterodimeric complex consisting of two subunits: the subunit CA, also called Crotapotin, and the subunit CB, also called Crotoxin B. The two subunits (CA/CB) are linked by non-covalent bonds [1,2]. The subunit CA is not toxic, but the subunit CB generally has PLA_2_ activity and is toxic to mammals. The lethality of crotoxin is favored and increased with the formation of the complex CA/CB [3,4]. Regarding the CB subunit, it acts at the neuromuscular junction level, causing inhibition in the release of acetylcholine [5]. In in vitro tests, CB depolarizes skeletal muscle by increasing creatine kinase release and producing myonecrosis [6]. Although the CB subunit blocks neuromuscular transmission, the mechanism of action has not been fully described [4]. The subunit CB has some conserved regions, such as the His48, Asp49, Tyr52, and Asp99 residues, which play an important role in the active site for phospholipid hydrolysis [6]. Several homologous proteins to the subunit CB have been described, and many of them vary in their toxicity and enzymatic activities [2,7]. Some viper venoms such as *C. s. scutulatus*, *C. tigris*, *C. vengrandis*, *C. horridus*, *C. simus*, *C. tzabcan*, *Bothriechis negroviridis*, and *Ophryacus sphenophrys* also contain the crotoxin complex CA/CB, with CB being the one with the greatest toxic activity [8]. Crotoxin B or similar structures, usually named crotoxin-like proteins, may represent a large percentage in the *Crotalus* spp. venoms, ranging from 4.3% in *C. basiliscus* to about 60% in *C. d. cumanensis* and *C. d. cascavella* [4,9].

Concerning *Crotalus* envenomation, crotoxin complex CA/CB and Crotamine are the main toxins associated with poisonings, besides the enzymatic activities of proteases in the crotalid venoms; therefore, the neutralization of these proteins, as well as some proteases, are key in the production process of commercial antivenoms against *Crotalus* spp. [10]. It is known that some commercial antivenoms have shown a lower affinity to the crotoxin complex or the CB region thereof when they have been compared with antivenoms obtained by immunization only with Crotoxin (CA/CB) or with only the CB subunit [11,12]. Thus, the neutralizing capacity of commercial antivenoms, for example, against *C. durissus* venom, rests mainly on the neutralization of crotoxin [13]. Recent studies have sought to improve antivenoms by enriching venoms with native CB [14]. That is, Fusco et al., (2015) [14] have proposed a new model to produce antibodies against *Crotalus* venoms. They administered the purified Crotoxin B first and then the complete venom. Their results showed that this model generated a greater number of antibodies against crotoxin than the traditional method of immunization (using the complete venom from the beginning), as well as an improvement in the neutralization of the toxicity of the venoms. Since a variety of our studies have focused on generating recombinant proteins as immunogens to develop a greater number of neutralizing antibodies [15,16], we here focused in on the recombinant expression of a Crotoxin B to evaluate its capacity as an immunogen and its ability to produce neutralizing antibodies against crotalid venoms. Although the production of a recombinant Crotoxin B has been reported, the authors neither showed the amino acid sequence of the Crotoxin B expressed nor confirmed their expression by mass spectrometry [5]. Furthermore, nonbiological activity was reported for such recombinant protein [5]. Therefore, in this work, we performed the cDNA cloning and heterologous expression of a CB subunit from *C. tzabcan,* a large rattlesnake species, endemic to the Yucatán Peninsula in Mexico [17]. The *C. tzabcan* venom provokes hemorrhagic, dermonecrotic, and inflammatory-edematogenic effects in mice, and even deaths have been recorded by its bite [18]. Furthermore, the recombinant CB, named here HisrCrotoxin B, was used as an immunogen to raise anti-HisrCrotoxin B rabbit antibodies, which reduced the *C. tzabcan* PLA_2_ venom activity and neutralized the venoms of *C. tzabcan* and other *Crotalus* spp. Additionally, anti-HisrCrotoxin B antibodies could recognize native Crotoxin B from different *Crotalus* species and discriminate venom in species with high or low levels of or absence of Crotoxin B.

## 2. Results and Discussion

### 2.1. cDNA Isolation, Sequence Determination, and Cloning of Crotoxin B from Crotalus Tzabcan

Based on the N-terminal sequence of Crotoxin B from *Crotalus tzabcan*, oligonucleotides were designed (Table S1). The cDNA coding region for HisrCrotoxin B with the expected size (378 bp) was amplified by PCR and cloned into plasmid pCR2.1-TOPO^®^. The transcript that codes for Crotoxin B has 122 residues, including 14 cysteines that form 7 disulfide bonds, and it is 99% identical to the Crotoxin B from *C. durissus terrificus* (Table 1). Crotoxin B was subsequently cloned into an expression vector, which included 19 extra amino acids at its N-terminus (MRGSHHHHHHGSENLYFQG). The mature peptide contained the restriction site *Bam*HI (GS), a His-Tag region added by the pQE-30 vector, and the proteolytic TVE site (ENLYFQG), resulting in 141 residues of the His-Tagged neurotoxin (Figure S1). The construction was named HisrCrotoxin B, and it retains the conserved residues His48, Asp49, Tyr52, and Asp99 of the common PLA_2_ active site (here, His47, Asp48, Tyr51, and Asp89), which were agreed to be characteristic of type II phospholipases [19] (Table 1).

### 2.2. Expression, Purification, and Protein Folding of HisrCrotoxin B

**Figure 1:**
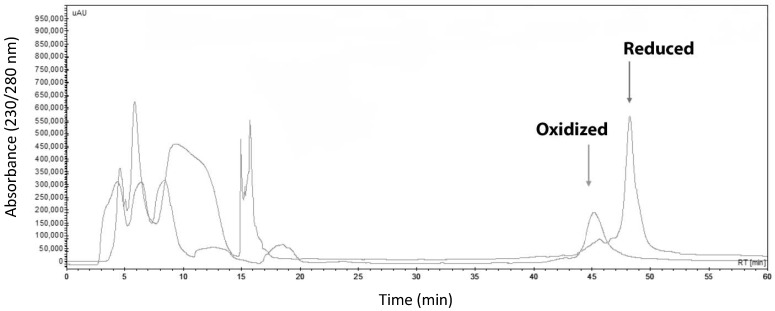
Overlapping HPLC profiles of the oxidized and reduced HisrCrotoxin B. After purification from nickel affinity, HisrCrotoxin B was reduced with DTT and separated by HPLC. The reduced HisrCrotoxin B had a retention time of 48 min. After folding, the oxidized HisrCrotoxin B was separated by HPLC. The oxidized HisrCrotoxin B had a retention time of 44 min. The HPLC separation was performed using a C18 column in a gradient of 0 to 60% of solution B in 60 min, where solution B is 0.1% trifluoroacetic acid (TFA) in acetonitrile.

**Figure 2:**
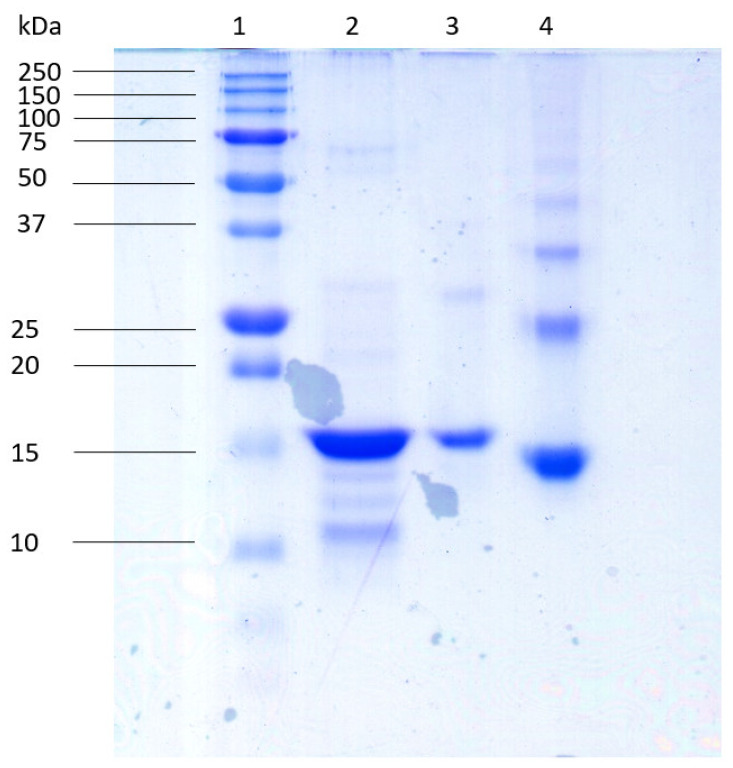
Profile of the recombinant and native protein in acrylamide gel at 15%. (1) Molecular weight marker; (2) reduced HisrCrotoxin B protein; (3) folded recombinant protein; (4) native Crotoxin B protein of *C. tzabcan*. A total of 10 µg of each sample reduced with β-mercaptoethanol was used.

The heterologous expression of HisrCrotoxin B was achieved using the *E. coli* Origami strain. HisrCrotoxin B was found mainly in inclusion bodies, and it was recovered after using agarose nickel affinity. HisrCrotoxin B was reduced with 1,4-dithiothreitol (DTT), and it was purified using a high-pressure liquid chromatography (HPLC) system using reverse-phase chromatography (RP-HPLC). The protein fraction with a retention time of 48 min was collected (Figure 1). An analysis by 15% SDS-PAGE showed that the protein fraction had a similar apparent molecular mass to the expected molecular mass (Figure 2).

Furthermore, the protein fraction showed an experimental molecular mass of 16,421.7 Da, obtained by mass spectrometry. It corresponded to the expected molecular mass for the 6His-tagged HisrCrotoxin B in its reduced form. The protein yield of HisrCrotoxin B was 1 mg/L. After purification by HPLC, the protein was folded in vitro following the conditions used by Fonseca et al. [20]; that is, the folding reaction contained 12 µM HisrCrotoxin B, 0.05 mM Tris-base, 2 M GndHCl, 1 mM CaCl_2_, and 1.7:0.2 mM Cys:Cys-Cys as a par redox, pH 6.0. The solution was continuously stirred for 48 h at room temperature. After that, the solution was centrifugated for 10 min at 16,000× *g* in an Eppendorf Centrifuge 5418 and purified again by RP-HPLC using the abovementioned same conditions. HisrCrotoxin B, in its oxidized form, eluted at a retention time of 44 min (Figure 1).

### 2.3. Evidence of Similar Structure between Native Crotoxin B and HisrCrotoxin B

**Figure 3:**
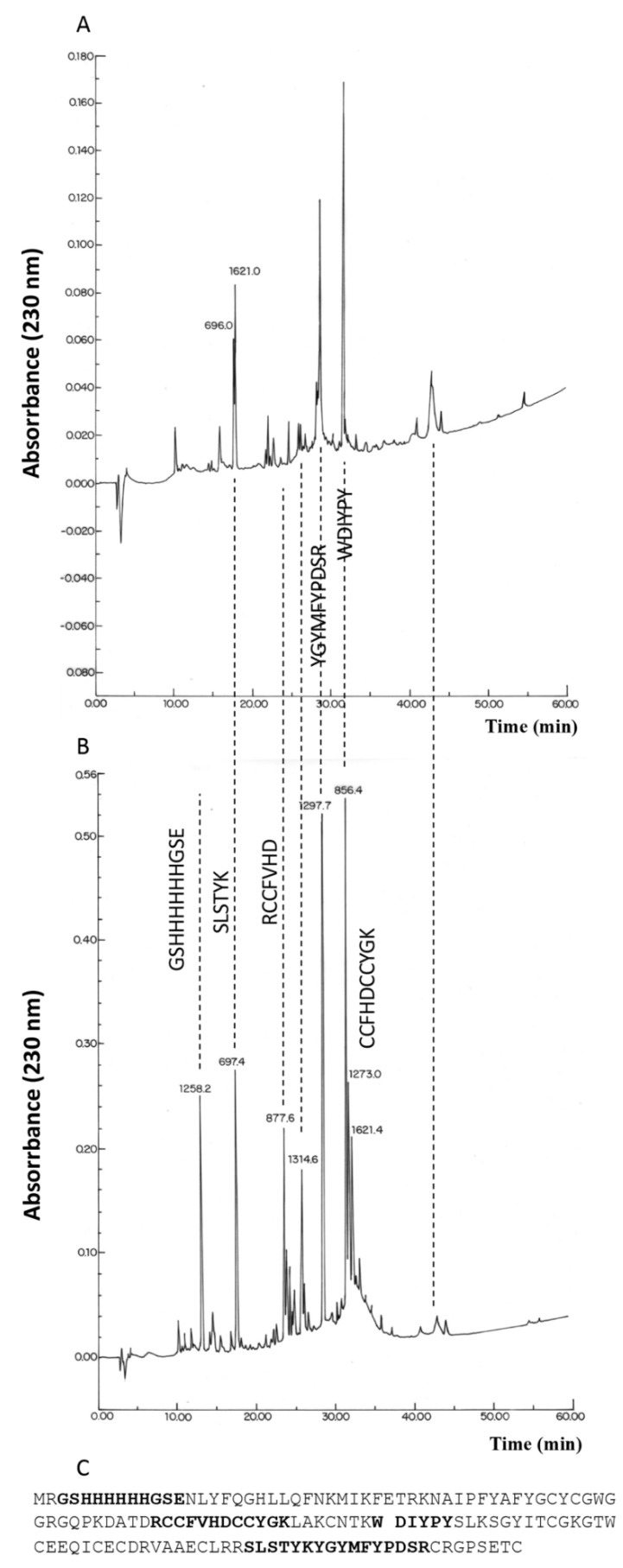
Reverse-phase HPLC profiles of the enzymatically digested native Crotoxin B (nCrotoxin B) and HisrCrotoxin B. Both nCrotoxin B (**A**) and HisrCrotoxin B (**B**) were enzymatically digested with trypsin and Glu-C. The amino acid sequences in (**A**) and (**B**) were found in the digested fractions. The digested peptide fractions were separated using an analytic C_18_ reverse-phase column (Vydac 214 TP 4.6 × 250 mm, Columbia, MD, USA) using 0.1% trifluoroacetic acid (TFA) in water as solvent A, and 0.1% TFA in acetonitrile as solvent B. The gradient was run from 20 to 60% solvent B for 40 min at 1 mL/min, and the peptide fragments were detected at 230 nm. The HPLC fractions collected were analyzed using mass spectrometry and Edman degradation. (**C**) The amino acid sequence of HisrCrotoxin B shows in bold the amino acid sequences detected by mass spectrometry and Edman degradation in the digested fractions.

It is important to note that a heterologously expressed protein containing 14 cysteines could theoretically form up to 135,135 isoforms if considering only the disulfide pairing possibility. The number of possible Cys-Cys structural forms for a protein rich in cysteines increases with the number of cysteines in the molecule; consequently, to obtain a structural enduring and in vivo functional recombinant protein, the right structure must be preserved. The oxidized HisrCrotoxin B and the native Crotoxin B (nCrotoxin B) were enzymatically digested, first with trypsin and then with Glu-C, to compare their structures. The digested protein fractions were separated by RP-HPLC, and the most prominent peptide fractions were analyzed by mass spectrometry and Edman degradation. The HPLC elution profiles displayed digested fraction patterns with similar retention times having the same molecular masses, yielding the same amino acid sequences for both the native Crotoxin B and the HisrCrotoxin B (Figure 3). If different HPLC elution profiles were displayed for any of the two proteins, it could indicate a different disulfide pairing arrangement.

**Figure 4:**
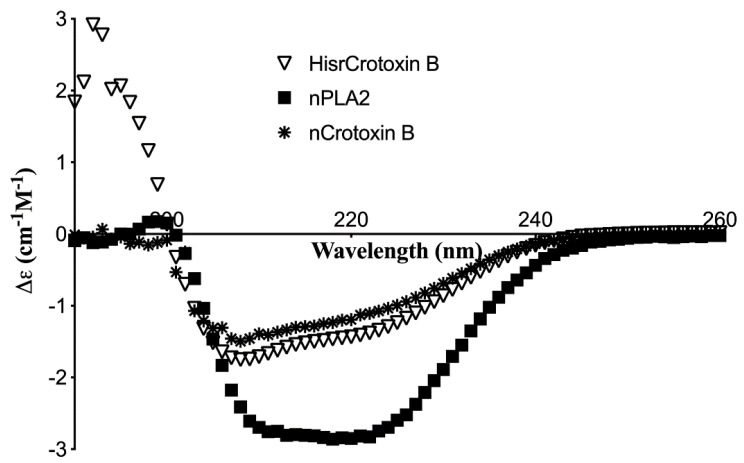
Circular dichroism of HisrCrotoxin B, native Crotoxin B, and native PLA_2_. The secondary structure analysis revealed a higher content of β-structures for HisrCrotoxin B and native Crotoxin B. The native PLA_2_ was isolated from *Bothrops ammodytoides* venom [15,16].

Furthermore, to compare the secondary structure of HisrCrotoxin B, the native Crotoxin B from *C. tzabcan* and a native PLA_2_ that was isolated from *Bothrops ammodytoides* venom were analyzed by circular dichroism (CD). The subunits, HisrCrotoxin B and the native Crotoxin B, showed high absorption for β-structures and relatively low content for α-helix secondary structure (Figure 4). According to a CD deconvolution program (BeStSel, https://bestsel.elte.hu, (accessed on 5 May 2022), the secondary structure contents were 13.6, 37.0, 18.5, and 30.9; 9.5, 28.3, 15.7, and 45.4; and 19.7, 22.7, 15.7, and 41.9 of α-helix, β-structures, β-turns, and other structures, respectively, for HisrCrotoxin B, native Crotoxin B, and nPLA_2_, respectively. So far, all pit viper venom PLA_2_s were found to contain a greater proportion of α-helix, characterized by a positive band at 198 nm and negative ellipticities at 208–222 nm [21].

In 1989, Aird et al. [22] revealed comparative spectroscopic studies of four crotoxin homologs and their subunits. They examined the secondary structures of four native crotoxins and their purified subunits from the venoms of *C. durissus terrificus, C. vegrandis, C. s. scutulatus*, and *C. viridis concolor* by circular dichroism. The obtained CD spectra of the isolated subunits of Crotoxin B were decreased slightly in α-helix, while they were increased in β-sheet structures, relative to intact native crotoxins [22]. For example, the native subunit Crotoxin B from *C. durissus terrificus* had 16, 51, and 33% of α-helix, β-sheet structure, and other structures, respectively. In this work, according to the CD deconvolution program used (BeStSel), the secondary structure contents of the native Crotoxin B were 9.5, 28.3, 15.7, and 45.4 of α-helix, β-structures, β-turns, and other structures, respectively. The secondary structures observed for all four native Crotoxin B by Aird et al. [22] are quite similar to the native Crotoxin B from *C. tzabcan* and the recombinant HisrCrotoxin B. Contrary to the CD of the subunits of Crotoxin B (native and recombinant), the intact native Crotoxin from *C. durissus terrificus* had 29, 23, and 48% of α-helix, β-sheet structure, and other structures [22], respectively, which had higher α-helix and lower content of β-sheet structure values like the control used (PLA_2_ from the viperid *B. ammodytoides*). Comparing the low content of α-helix of HisrCrotoxin B and the native Crotoxin B obtained here by CD with that of the α-helix obtained by X-ray crystallography of native Crotoxin B [2,23,24,25] and other PLA_2_s, they may differ in the fact that the X-ray crystallography structures are practically obtained in a semi-solid/solid state, maximizing the hydrogen bonds of the secondary structures. On the other hand, the secondary structure obtained by CD is obtained in a more dynamic liquid environment; however, the CD deconvolution algorithms are based on few minima of wavelength light absorption, such as 208 and 222 nm, and in the number of other proteins in the CD database used. Therefore, the content of α-helix secondary structure obtained by CD or X-ray crystallography may have some deviations. To have a better comparison, in this work, we compared the CD of both HisrCrotoxin B and the native Crotoxin B, and also with a relate native PLA_2_, which has a slightly higher content of α-helix, but still lower than that of other viperid PLA_2_s such as that of BmajPLA2-II, a basic Lys49 PLA_2_ homologue [26].

### 2.4. Rabbit Immunization, Antibody Recognition, Antibody Titers, and Inhibition of Phospholipase Activity

**Figure 5:**
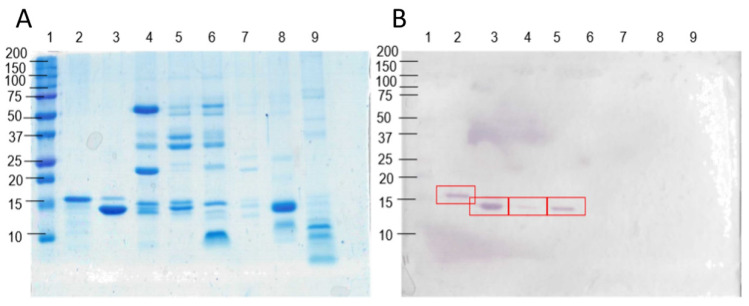
SDS-PAGE and Western blot, under reducing conditions, of native crotoxins and different animal venoms. (1) Molecular weight markers; (2) HisrCrotoxin B; (3) native crotoxin from *C. tzabcan*; (4) *C. tzabcan* venom; (5) *C. mictlantecuhtli* venom; (6) *C. culminatus* venom; (7) *Bothrops asper* venom; (8) *Micrurus laticollaris* elapid venom; (9) *Chihuahuanus crassimanus* scorpion venom. (**A**) SDS-PAGE and (**B**) Western blot where the red marks show recognition of anti-Crotoxin B rabbit antibodies to native crotoxin and venoms from *C. tzabcan* and *C. mictlantecuhtli.* The antibody recognition was observed in proteins ranging from 10 to 15 kDa, which represent the native crotoxins from *Crotalus* spp. venoms.

The rabbits were immunized each with 7.3 mg of HisrCrotoxin B. After 3 months of immunization, the rabbits were bled, and the serum was used to observe its capacity to recognize HisrCrotoxin B and native crotoxins from *Crotalus* spp. venoms. Figure 5A shows the protein composition of venoms from several species of *Crotalus*. Native crotoxins, according to their observed apparent molecular masses, might have from none to one-third of their respective venom composition (see red square marks in Figure 5B). The apparent molecular mass of HisrCrotoxin B (Figure 5B, lane 2) looked larger than the other natural crotoxins. This may have been caused by the extra basic residues (6His-tag) at the N-terminal of HisrCrotoxin B, which might retard the protein migration, as observed previously [27]. Nevertheless, the mass spectrometry confirmed the expected molecular mass for HisrCrotoxin B. Yet, Figure 5B shows the recognition of the rabbit anti-HisrCrotoxin B to venom proteins from *Crotalus* species (*C. tzabcan* and *C. mictlantecuhtli*), including native Crotoxin B. However, it did not recognize venom proteins from *C. culminatus, Bothrops asper*, *Micrurus laticollaris* (elapid), and *Chihuahuanus crassimanus* (scorpion). The antibody recognition was observed in proteins from 10 to 15 kDa, representing the native crotoxins from *Crotalus* spp. venoms, but *C. culminatus* did not contain Crotoxin B [28].

**Figure 6:**
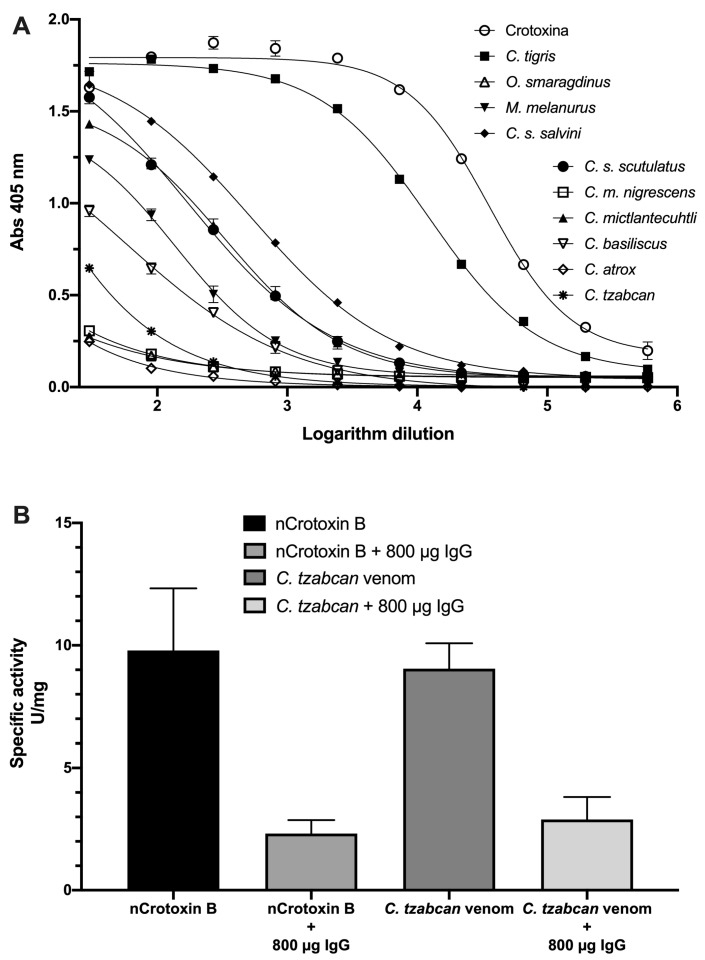
Antibody recognition of rabbit anti-HisrCrotoxin B to venoms from *Crotalus* species (*n* = 2). (**A**) Anti-HisrCrotoxin B recognition towards venoms from different species of *Crotalus*. (**B**) Inhibition of the phospholipase activity of *C. tzabcan* venom by anti-HisrCrotoxin B rabbit antibodies (*n* = 4).

The recognition of Crotoxin B within the viperid venoms by anti-HisrCrotoxin B was evaluated in ELISA plates to support the previous observations (Figure 6A). The IC_50_ values represented the serum concentration (µg/mL) required to obtain half of the colorimetric response. They had implicit the concentration of an inhibitor where the response (or binding) was reduced by half (Table 2). It is important to note that ELISA experiments allow the recognition of structural epitopes. In other words, the titers represent the needed concentration of antibodies (IC_50_ in µg/mL) to recognize structural similarities in Crotoxin B within the viperid venoms. Table 2 summarizes the IC_50_ values. The venoms *C. tigris*, *C. s. salvini*, and *C. mictlantecuhtli* had the best IC_50_ values (lower µg/mL). *M. melanurum*, *C. s. scutulatus*, and *C. basiliscus* had significant recognition of Crotoxin B, but *C. atrox*, *C. tzabcan*, *O. smaragdinus*, and *C. m. nigrescens* had poor recognition (higher µg/mL) of Crotoxin B.

Moreover, the anti-HisrCrotoxin B antibodies were tested against the phospholipase activity of *C. tzabcan*. Therefore, different concentrations of anti-HisrCrotoxin B were pre-incubated for one hour at 37 °C with 15 µg of the native protein. Once such a time had elapsed, the enzymatic activity assay was carried out by a conventional titrimetric assay. As mentioned previously, the phospholipase activity of the native Crotoxin B was determined at 9.8 ± 2.5 U/mg. In comparison, the activity of the same pre-incubated protein plus 800 µg of anti-HisrCrotoxin B antibodies was 2.3 ± 0.5 U/mg, representing only 23% of original PLA_2_ activity. In the same way, the enzymatic activity was performed with the complete venom of *C. tzabcan*, which had an activity of 9 ± 1 U/mg, while the PLA_2_ activity of the whole venom pre-incubated plus 800 µg of anti-HisrCrotoxin B antibodies was 2.9 ± 0.9 U/mg, representing 32% of the original phospholipase activity of *C. tzabcan* venom (Figure 6B).

### 2.5. Neutralization Activity

Although the antibody recognition, by both Western blot and ELISA plates, and inhibition of phospholipase activity were evident, in the titimetric assay, towards Crotoxin B in crotalid venoms, the neutralization assays are needed to assess the relevance of HisrCrotoxinB as an immunogen. For that, an in vivo neutralization test was performed using mice (CD1, 18–20 g). Three median lethal doses (LD_50_) of native Crotoxin B and crotalid venoms were challenged against different concentrations of anti-HisrCrotoxin B pre-incubated at 37 °C for an hour in a final volume of 200 µL with PBS, which were administered intravenously to the mice. Their survival was evaluated at 48 h after administration. 3LD_50_ assessed with pre-immune antibodies or without anti-HisrCrotoxin B were used as a positive control, also incubated for an hour at 37 °C. Similarly, anti-HisrCrotoxin B against the complete venoms of *C. tzabcan, C. s. salvini*, and *C. miclanthecuhtli* were challenged using the same protocol. Table 3 shows the neutralizing potency, expressed as mean effective dose (ED_50_) against native Crotoxin B, which was 1.5 mg/3LD50, while the ED_50_s against the complete venoms of *C. tzabcan, C. s. salvini*, and *C. miclanthecuhtli* were 1.5, 14, and 2.1 mg/3LD50, respectively. Thus, the venom neutralization was effective for venoms of *C. tzabcan, C. s. salvini*, and *C. miclanthecuhtli*. Although ELISA assays are far from being able to be used to predict antivenom neutralization, interesting data can be inferred from them. For example, the antibody recognition experiments were correlated with the neutralization assays. That is, the venoms of *C. tzabcan, C. s. salvini*, and *C. mictlantecuhtli* were recognized and neutralized (Table 3).

These data proved that HisrCrotoxin B could be a suitable immunogen to raise anti-Crotoxin B and anti-PLA_2_ antibodies to decrease PLA_2_ activity of *C. tzabcan* phospholipases, related to several toxic activities such as myotoxicity or interfering with the platelet function, at least in this *C. tzabcan* venom.

### 2.6. Irregular Presence of Crotoxin B in Crotalid Venoms

**Figure 7:**
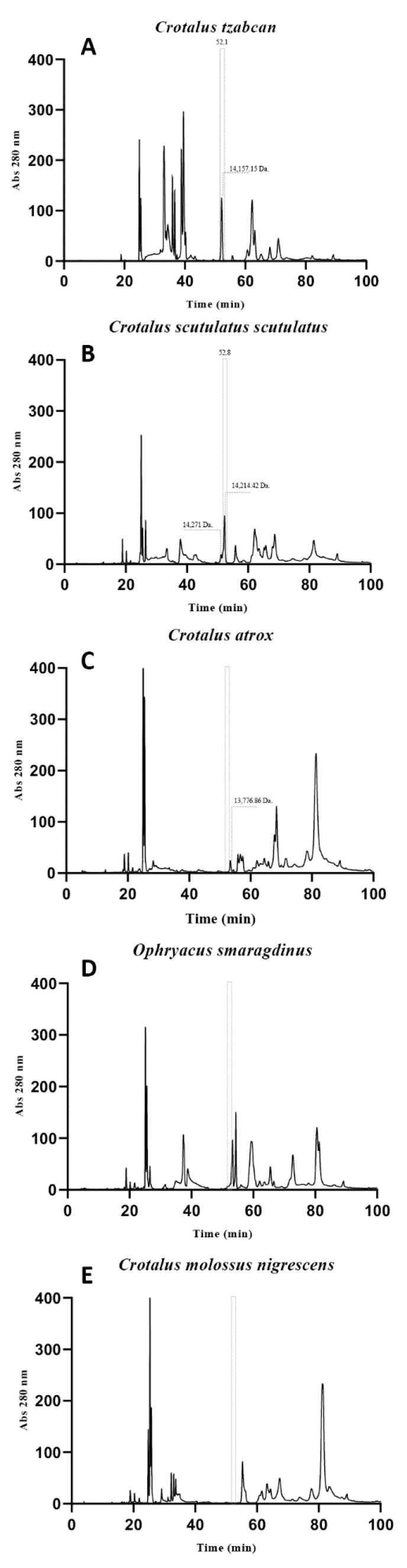
Cromatograms of selected crotalid venoms. (**A**) *C. tzabcan*, (**B**) *C. s. scutulatus*, (**C**) *C. atrox*, (**D**) *Ophryacus smaragdinus*, and (**E**) *C. m. nigrescens.* Fractionation corresponds to 1 mg of each venom using the HPLC-RP system (C18 Supelco^®^ Discovery^®^ column 25 cm × 4.6 mm, 5 µm, Merck KGaA, Darmstadt, Germany) with a gradient from 0 to 100% Sol B (Acetonitrile + 0.1% TFA) for 90 min).

Although Crotoxin B is a characteristic component of the venom of crotalids, it was noticed in Western blots and the ELISA assays that some crotalid individual venoms such as *C. atrox* and *C. m. nigrescens* did not seem to contain Crotoxin B. To determine the absence of Crotoxin B in some crotalid species, SDS-PAGE and Western blots were performed (Figures S2–S10). Moreover, a milligram of some crotalid pools of venoms was run by HPLC under the same conditions to compare retention times related to Crotoxin B from the venom of *C. tzabcan*. Figure 7 shows the RP-HPLC chromatograms of the whole venoms of selected viperid species *C. tzabcan*, *C. s. scutulatus*, *Ophryacus smaragdinus*, *C. atrox*, and *C. m. nigrescens.*

The venom from *C. scutulatus scutulatus* (52.8 min) contained a fraction with retention time and molecular mass (14,214.4 Da) like that of the venom from *C. tzabcan* (52.1 min), from which Crotoxin B was isolated. However, the venoms from *Ophryacus smaragdinus*, *C. atrox*, and *C. m. nigrescens* did not contain relevant fractions within such retention times. The molecular mass of 13,776.8 Da found for a fraction from *C. atrox* venom seemed to be a related PLA_2_ with domains perhaps different from that of Crotoxin B. The venoms of *O. smaragdinus* and *C. m. nigrescens* contained fractions with retention times later than that of the 52.1–52.8 min; however, no molecular masses were found for such fractions. Variation in the venom composition over a species’ geographic distribution is an integral part of intraspecific variation [28,29,30]. That is, venom composition variation in biological and biochemical activities among adults, as well as ontogenetic changes from juvenile to adults, has been observed. For example, juvenile venoms are more lethal and had higher percentages of crotamine and crotoxin than adults [31]. Moreover, it is known if diet, temperature, oxygen levels, humidity, and light cycles could affect such venom variation. Therefore, geographical differences may affect clinically because envenomations could present different symptomatology depending on the region. It is also important to know whether there is a geographic variation in the venoms used as immunogens because this might affect the antivenom quality [32]. Consequently, this work provides information to improve the quality of immunogens.

## 3. Conclusions

This work presents for the first time the use of a recombinant Crotoxin B subunit, HisrCrotoxin B, to produce rabbit serum antibodies against native crotoxins from *Crotalus* spp. venoms. The inhibition of PLA_2_ activity, the circular dichroism spectrum of HisrCrotoxin B, and the neutralization of *Crotalus* spp. venoms suggest that the recombinant Crotoxin B keeps basic secondary structure domains, such as native Crotoxin B, making it possible to raise neutralizing antibodies. Therefore, HisrCrotoxin B could be used as an immunogen to raise antibodies against related *C. tzabcan* species. The use of recombinant toxins as immunogens to raise neutralizing antibodies against simple neurotoxic venoms such scorpions (*Centruroides, Tityus, Androctonus*) and spiders (*Loxoceles*) has already been reported [33,34]. In the same way, other recombinant proteins could be used to generate neutralizing antibodies to reduce the toxic activities caused by the envenomation of poisonous animals. Furthermore, the reactivity of the anti-HisrCrotoxin B on other *Crotalus* venoms provides an interest in the study on the possible neutralization of toxic activities related to this toxin. Although currently anti-snake venom production for therapeutics are polyclonal antibodies produced in animals mostly using the whole venom as the immunogen, new methodologies are under study [35]. The use of recombinant proteins to generate polyclonal and monoclonal antibodies or neutralizing fragments through molecular biology methods could be the future for antivenoms production [36]. Moreover, snake venom PLA_2_s such as Crotoxin B can be used as an experimental model for the development of anti-inflammatory drugs for therapy in humans [37]. Therefore, this report provides a proof of principle for taking advantage of recombinant immunogens for developing commercial antivenoms or drug leads.

## 4. Materials and Methods

### 4.1. Venom and Venom Gland

Venoms were obtained from the Instituto de Biotecnología and from the Herpetario Kiinam (register number DF-REP-208-10-08, Cd. de Mexico, Mexico). An adult specimen of *Crotalus tzabcan* was kept in good health conditions and in plastic cages at 27 °C of constant temperature. The snake was fed fortnightly with a mouse, and tap water was provided ad libitum. The light-dark cycles were 12 h. The venom collected by manual extraction was immediately vacuum dried and stored at −20 °C until use. A healthy specimen was selected to obtain one of the two venom glands and removed by surgical extraction after being anesthetized with ketamine–xylazine. Immediately after extraction of the venom gland, it was treated with RNAlater^®^ (Thermofisher, Asheville, NC, USA) and stored at −20 °C until use. After surgical intervention, the specimen recovered itself and remained healthy.

### 4.2. Bacterial Strains, Enzymes, and Plasmids

The XL1-Blue *Escherichia coli* strain was used for DNA cloning and plasmid propagation. The Origami *E. coli* strain was employed to express the recombinant PLA_2_. Plasmids pCR^®^2.1-TOPO^®^ (Invitrogen, Carlsbad, CA, USA) and pQE30 (Qiagen, Valencia, CA, USA) were used for cloning the Crotoxin B gene and production of the 6His-tagged recombinant HisrCrotoxin B, respectively. Restriction enzymes, *Taq* polymerase, and T4 DNA ligase were purchased from New England Biolabs (New England Biolabs, Ipswich, MA, USA).

### 4.3. RNA Extraction and Gene Assembly

The total RNA was extracted from a single venom gland of *Crotalus tzabcan* using the “Total RNA Isolation System” (Qiagen, Valencia, CA, USA). Based on the N-terminal sequence of the previously reported PLA_2_, specific oligonucleotides were designed to amplify the corresponding transcript [35]. The oligonucleotides were named Oligo Crotoxin B-dir (GAG GGG CAC CTG CTG CAA TTC, Tm 61.4 °C) and Oligo Crotoxin B-rev (GAC TTA GCA TGT CTC TGA AGG CCC, Tm 59.3 °C).

The obtained insert amplified by *Taq* polymerase was purified using the High Pure PCR Product Purification Kit (Roche, Basel, Switzerland), then ligated to the plasmid pCR^®^2.1-TOPO^®^ (Invitrogen, Carlsbad, CA, USA), and finally used to transform quimiocompetent XL1-Blue *E. coli* cells. Positive clones were selected on the basis of the size of the amplified segment by colony PCR (M13-Forwar and M13-Reverse oligonucleotides). After plasmid purification (High Pure Plasmid Isolation Kit, Roche, Basel, Switzerland), the integrity of the gene construction was verified by DNA sequencing.

### 4.4. Plasmid Construction for Expression

The gene construction to express the HisrCrotoxin B included recognition sequences for restriction enzymes used for cloning (*Bam*HI and *Ps*tI) and a sequence encoding the TEV cleavage site. The designed transcript was subcloned into the pQE30 expression vector through the *Bam*HI and *Pst*I sites. The pQE30 vector introduces a polyhistidine-tag (6His) to facilitate product purification by affinity chromatography. The tobacco etch virus (TEV) cleavage sequence was conveniently placed between the 6His and the mature toxin to allow the cleavage of the whole recombinant toxin if necessary. The new pQE30-derived constructs were verified by sequencing from both sides. Quimiocompetent *E. coli* Origami cells were transformed with the corresponding plasmids by incubation for 30 min on ice, heat-shocked for 1 min at 42 °C followed by 5 min in ice, recovered for 1 h at 37 °C in SOC medium, and plated in LB containing 100 μg/mL of ampicillin. The constructions were named pQE30:HisrCrotoxin B, and their expression product was here abbreviated as HisrCrotoxin B.

### 4.5. Expression and Purification of HisrCrotoxin B

*E. coli* strain Origami expressing the plasmid pQE30:HisrCrotoxin B was grown in Luria Bertani (LB) broth. After the absorbance at 600 nm reached 0.8 of absorption units, the cultures were induced with 1 mM IPTG (isopropyl-β-D-thiogalactopyranoside) for 24 h at 16 °C. Cells were harvested by centrifugation (9800× *g* for 20 min in JA-14 rotor) using a Beckman centrifuge model J2-21, recovered in washing buffer (0.05 M Tris-HCl, pH 8.0), and lysed with One-Shot *cell disruptor* (Constant Systems^®^, Daventry, UK) at a pressure of 30 kpsi. This material was centrifuged again at 17,000× *g* for 15 min (Beckman Coulter Avanti J-30I^®^, rotor JA–20, Brea, CA, USA), and the supernatant was discarded.

The insoluble fraction was rinsed twice with washing buffer and centrifuged again at 17,000× *g* for 15 min. The inclusion bodies included in the insoluble fraction were solubilized with the chaotropic agent guanidinium chloride (GndHCl) 6M in a Tris-base 0.05 M buffer (pH 8.0) to extract the recombinant PLA_2_. Then, it was centrifuged at 17,000× *g* for 25 min using a Beckman Coulter Avanti J-30I^®^, rotor JA–20 (Brea, CA, USA) to remove the insoluble material. The supernatant that contained the recombinant protein was purified by Ni-NTA (Ni-nitrilotriacetic acid) affinity column chromatography, following the manufacturer’s instructions (Qiagen, Valencia, CA, USA). Buffer A (6 M GndHCl in a 0.05 M Tris-base buffer, pH 8.0) and buffer B (6 M GndHCl in 0.05 M Tris-base buffer, containing 400 mM imidazole, pH 8.0). Buffer B was eliminated by a second purification step under reverse-phase HPLC (RP-HPLC). An analytic C_4_ reverse-phase column (Vydac 214 TP 4.6 × 250 mm, Columbia, MD, USA) was run using a two solvent system with solvent A composed of 0.1% trifluoroacetic acid (TFA) in water and solvent B composed of 0.1% TFA in acetonitrile. Fractions were eluted by lineal increase in solvent B from 10 to 60%, during 50 min at 1 mL/min, and the proteins were detected at 230 nm. The HisrCrotoxinB product was vacuum dried. The recombinant product was allowed to fold under controlled conditions using 0.05 mM Tris-base, 2 M GndHCl, 1 mM Ca_2_Cl, and 1.7:0.2 mM Cys:Cys-Cys as a par redox, pH 6.0, containing 1.7 mM of cysteine (Cys) and 0.2 mM of cystine (Cys-Cys).

### 4.6. Molecular Mass Determination of HisrCrotoxin B, Native Crotoxin B, and Peptides from the Enzymatic Digestions

The molecular mass identities of HisrCrotoxin B, Crotoxin B from *C. tzabcan* and other native Crotoxin B, and the enzymatically digested peptides were confirmed by mass spectrometry analysis. The protein fractions were reconstituted to a final concentration of 500 pmol/5 μL of 50% acetonitrile with 1% acetic acid and directly applied to a Thermo Scientific LCQ Fleet ion trap mass spectrometer (San Jose, CA, USA) using a Surveyor MS syringe pump delivery system. The eluate at 10 μL/min was split out to introduce only 5% of the sample into the nanospray source (0.5 μL/min). The spray voltage was set from 1.5 kV, and the capillary temperature was set to 150 °C. The fragmentation source was operated at 25–35 V of collision energy and 35–45% (arbitrary units) of the normalized collision energy, and the scan with a wide band was activated. All spectra were obtained in the positive-ion mode. The data acquisition and deconvolution were performed on the Xcalibur Windows NT PC data system (Thermo Fisher Scientific, Waltham, MA USA).

### 4.7. Enzymatic Digestions of HisrCrotoxin B and Native Crotoxin B

Either Crotoxin B from *C. tzabcan* or recombinant HisrCrotoxin B were separately digested and incubated with trypsin (4 μg/mL at 37 °C) for 16 h. Afterward, Glu-C was added (2.5 μg/mL at 37 °C) for 6 h more. The digested peptides were fractionated using an analytic C_18_ reverse-phase column (Vydac 214 TP 4.6 × 250 mm, Columbia, MD, USA) running from solvent A (0.1% trifluoroacetic acid, TFA, in water) to solvent B (0.1% TFA in acetonitrile). The HPLC system previously described was used for this separation, and the gradient was run from 0 to 60% solvent B for 60 min at 1 mL/min; the proteins were detected at 230 nm. Additionally to the mass spectrometry analysis of the digested peptides (Section 4.6), N-terminal Edman degradation was performed on a Shimadzu PPSQ-31A (Shimadzu, Kyoto, Japan) automated gas-phase sequencer. A sample (60 µg) was dissolved in 10 mL of 37% CH3CN (*v*/*v*) solution and applied to TFA-treated glass fiber membranes, pre-cycled with Polybrene (Sigma-Aldrich Co. St. Louis, MO, USA).

### 4.8. Circular Dichroism

The secondary structure contents of a native PLA_2_ and recombinant HisrCrotoxin B were calculated using circular dichroism spectroscopy (CD). Spectra were recorded at room temperature in quartz cells (1 mm-path) and wavelengths from 190 to 260 nm using a spectropolarimeter Jasco J-710 (Jasco, Japan). Data were collected every 1 nm at 50 nm/min. Each protein was dissolved in 60% trifluoroethanol up to a 0.6 mg/mL concentration. Trifluoroethanol improves secondary structure. The CD values correspond to the mean of three CD recordings. Finally, percentages of secondary structure were analyzed using the algorithms hosted online at BeStSel (Beta Structure Selection. webserver http://bestsel.elte.hu/index.php (accessed on 5 May 2022) [38].

### 4.9. Electrophoretic Analysis and Western Blotting

For electrophoretic analysis, SDS-PAGE under reducing conditions (4/15%) was performed according to the method proposed by Laemmli [39]. The protein staining was made with Coomassie Brilliant Blue. For Western blotting analysis, venom samples were first separated by SDS-PAGE (15%) and then transferred to a polyvinylidene difluoride membrane using a transfer apparatus Owl semi-dry system, 1 h at 400 mA. After transference, the membrane was incubated with 5% nonfat milk in TBST (10 mM Tris (pH 8.0), 150 mM NaCl, 0.5% Tween 20) for 2 h at room temperature, then washed three times with TBST and incubated with the obtained rabbit IgG antibodies (1:50) for 1 h, rewashed three times with TBST, and finally incubated with the second antibody (goat anti-rabbit IgG coupled to alkaline-phosphatase at 1:2000). The membrane was washed with TBST three times and developed with 3,3′,5,5′-tetramethylbenzidine (TMB) ready-to-use solution (Invitrogen Antibodies, Thermo Fisher Scientific, Asheville, NC, USA) according to the manufacturer’s protocols.

### 4.10. Animal Immunizations

Rabbits were hyperimmunized subcutaneously with 7.3 mg of HisrCrotoxin B to raise serum antibodies (anti-HisrCrotoxin B). The immunization protocol started with 0.02 mg total protein in Complete Freud’s Adjuvant (CFA). Increasing doses up to 0.64 mg, alternating between incomplete Freud’s (IFA) and aluminum hydroxide (AH), were applied for 5 months, besides 4 boost applications without adjuvant injected every month along with the immunization. The rabbit serum antibodies were purified from plasma by acid precipitation using 5% caprylic acid.

### 4.11. Enzyme-Linked Immunosorbent Assay (ELISA)

Solid-phase-adsorbed MaxiSorp plates (NUNC™, Thermo scientific, Waltham, MA, USA) were prepared by treating wells with a 100 µL solution of 5 µg/mL of each protein (HisrCrotoxin B, native Crotoxin B, and venoms) in 100 mM sodium carbonate buffer (pH 9.6). After overnight incubation at 4 °C, the wells were aspirated and washed three times with 200 µL of a washing buffer (50 mM Tris-HCl (pH 8), containing 0.5 mg/mL of Tween 20 and 150 mM NaCl). The wells were then filled with 200 µL of blocking buffer (5 mg/mL gelatin and 2 mg/mL Tween 20 in 50 mM Tris-HCl buffer at pH 8). After 2 h incubation at 37 °C, the wells were washed, as described above, and filled with 100 µL of serially diluted rabbit IgG anti-HisrCrotoxin B in incubation buffer (50 mM Tris-HCl buffer at pH 8 containing 1 mg/mL gelatin, 0.5 mg/mL Tween 20, and 0.5 M NaCl). The starting dilution was 1:30, from an IgG concentration of 4 mg/mL, and the incubation time was 1 h at 37 °C. After washing, the bound rabbit IgGs were allowed to react with 100 µL per well of 0.1 mg/mL of anti-rabbit IgGs labeled with horseradish peroxidase (Roche, Basel, Switzerland) in the incubation buffer. After 1 h at 37 °C, wells were washed and filled with 100 µL of 2,2’-azinobis [3-ethylbenzothiazoline-6-sulfonic acid]-diammonium salt (ABTS) solution (Roche, Gaithersburg MD, USA) as the substrate for peroxidase. The color development reaction was stopped by adding 25 µL of 20% sodium dodecyl sulfate (SDS), and the plate was read at 405 nm in a Microplate Reader (Tecan Sunrise IVD version, Chapel Hill, NC, USA). Data were analyzed by nonlinear regression using the sigmoidal dose–response equation of the Prism program (Graph Pad Prism v. 6.0c, San Diego, CA, USA). Conventional titers were calculated from the midpoint of the curve and correspond to the IgG dilution for half of the maximal binding, and it was considered the half-maximal inhibitory concentration (IC_50_).

### 4.12. Phospholipase Activity

The hydrolysis of egg yolk at 10% determined the phospholipase activity of the purified enzyme in a buffer (0.1 M NaCl, 0.01 M CaCl_2_, 0.5% Triton X-100). The buffer’s pH was set to 8.05, and the sample was added. We measured the time when the pH value dropped to 7.99, and then 0.05 M NaOH was used to up the pH value again. We continued measuring the time when the pH value dropped again in a total of 5 repetitions. Slight N_2_ bubbling was applied during the assay to avoid the O_2_ intervention in the reaction. The activity was determined as the µmol of NaOH consumed per minute per mg of protein.

### 4.13. Neutralization Activity

The protocol used for assaying the activity of HisrCrotoxin B in vivo, using a mouse model, was followed according to our Institute Committee of Animal Welfare guidelines, keeping the number of animals to minimum required to validate the experiments. Male mice (CD-1, 18–20 g body weight) were tested by intravenous injection.

### 4.14. Statistics

Results were expressed as mean and standard deviation or as mean with 95% confidence intervals. The software Prism 4.0 (Graph Pad Inc., San Diego, CA, USA) was used for all statistical methods.

## Figures and Tables

**Table 1:** Amino acid sequence of HisrCrotoxin B compared to the native Crotoxin B.

Protein	Amino Acid Sequence *	ID (%)
	1--------10--------20--------30--------40-------50---------61	
Crotoxin B	HLLQFNKMIKFETRKNAIPFYAFYGCYCGWGGRG*R*PKDATDRCCFVHDCCYGKLAKCNTKW	100
HisrCrotoxin B	HLLQFNKMIKFETRKNAIPFYAFYGCYCGWGGRG*Q*PKDATDRCCFVHDCCYGKLAKCNTKW	99
	**********************************:**************************	
	62------70--------80--------90-------100------110---------122	
Crotoxin B	DIYPYSLKSGYITCGKGTWCEEQICECDRVAAECLRRSLSTYKYGYMFYPDSRCRGPSETC	100
HisrCrotoxin B	DIYPYSLKSGYITCGKGTWCEEQICECDRVAAECLRRSLSTYKYGYMFYPDSRCRGPSETC	99
	*************************************************************	

* The first line represents the position numbers to locate the position of residues in the amino acid sequences. Residues in cursive differ in Crotoxin B (from *Crotalus durissus terrificus*, P62022.1, the first characterized Crotoxin B) and HisrCrotoxin B. The conserved underlined residues H47, D48, Y51, and D89 may represent the active site of the PLA_2_ activity. The cysteine residues are in bold. Asterisks represents identical amino acids, and the colon punctuation mark represents different amino acids in Crotoxin B and HisrCrotoxin B. ID means a percentage of identity.

**Table 2:** IC_50_ values for recognition of native Crotoxin B and viperid venoms by antibodies from anti-HisrCrotoxin B.

Protein/Venom	IC_50_ (µg/mL)	CI *
Native Crotoxin B	0.1	0.09–0.14
*C. tigris*	0.3	0.28–0.38
*C. s. salvini*	7.6	6.2–9.6
*C. mictlantecuhtli*	12.3	10.9–13.9
*C. s. scutulatus*	23.2	19.5–28.9
*M. melanurum*	29.1	25.0–35.4
*C. basiliscus*	70.2	42.2–165.5
*C. tzabcan*	910.4	265–4451
*O. smaragdinus*	>4000	nd
*C. m. nigrescens*	>4000	nd
*C. atrox*	>4000	nd

* CI means confidence intervals (95%); nd means not determined.

**Table 3:** Determination of the mean effective dose (ED_50_) of anti-Crotoxin B antibodies challenged against native Crotoxin subunit B and crotoxin-positive venoms.

Venom/Toxin	LD_50_ µg/mouse	ED_50_ mg/3LD_50_	Crotoxin B (%)	Potency (µg venom/mg AV)
Native Crotoxin B	6.7	1.5	100	13.4
*C. s. salvini*	4.7	14.0	8.9	1.0
*C. tzabcan*	19	1.5	7.7	38.0
*C. mictlantecuhtli*	4	2.1	10.3	5.7
*C. m. nigrecens*	59.2	>15	0	-

LD_50_: median lethal dose showing the dose of toxin or venom at which 50% of the mouse population die; ED_50_: the amount of anti-HisrCrotoxin B antibodies (mg) at which 50% of the mouse population survive against 3LD50 of toxin or venom; Potency: the amount of venom (µg) neutralized per milligram of anti-HisrCrotoxin B antibodies; Crotoxin B: the reported amount (%) of Crotoxin in the sample.

## Supplementary Materials

The following supporting information can be downloaded at https://www.mdpi.com/article/10.3390/toxins14060382/s1, Table S1: Design of oligos for Crotoxin B with restriction sites BamH1 and Pst1. Figure S1: Representation of the gene construction for the heterologous expression of HisrCrotoxin B. The primary structure of HisrCrotoxin B in bold. Figures S2–S10: SDS-PAGE and Western blot of crotalid venoms.
