# Supplementary material for: Crotoxin B: Heterologous Expression, Protein Folding, Immunogenic Properties, and Irregular Presence in Crotalid Venoms
Source: Toxins (Basel). 2022 May 31;14(6):382. doi: 10.3390/toxins14060382 (PMC9228539; doi:10.3390/toxins14060382)
Supplement: Supplementary file 1 [file toxins-14-00382-s001.zip › toxins-1710576-supplementary.pdf]

Supplementary Materials: Crotoxin B: Heterologous Expression, Protein Folding, Immunogenic Properties, and Irregular Presence in Crotalid Venoms

Miguel Angel Mejía-Sánchez, Herlinda Clement, Ligia Luz Corrales-García, Timoteo Olamendi-Portugal, Alejandro Carbajal and Gerardo Corzo

Table S1. Design of oligos for CrotoxinB with restriction sites BamH1 and Pst1

Description	oligonucleotides (5′ →3′)
HisrCrotoxinB -pQE30-Fwr Length 46 nt	GAGA *GGA TCC* GAA AAC CTG TAT TTT CAG GGT CAC CTG CTG CAA TTC
HisrCrotoxinB -pQE30-Rev Length 32 nt	TCTC CTG CAG **CTA TTA** GCA TGT CTC TGA AGG C

Site BamHI: *GGA TCC*; Site TEV: GAA AAC CTG TAT TTT CAG GGT; Site STOPs: **CTA TTA**; Site PstI: CTG CAG.

***Bam*H1**

-*PT*5-

*MRGSHHHHHHGSENLYFQG***HLLQFNKMIKFETRKNAIPFYAFYGCYCGWGGRGQPKDATDRCCFV HDCCYGKLAKCNTKWDIYPYSLKSGYITCGKGTWCEEQICECDRVAAECLRRSLSTYKYGYMFYP DSRCRGPSETC****LQ

***Pst*1**

Figure S1. Representation of the gene construction for the heterologous expression of HisrCrotoxinB. The primary structure of HisrCrotoxinB is in bold. The 6His-coding sequence is part of the pQE30 vector and is located upstream of the *Bam*HI/*Pst*I-cloned gene, so the recombinant protein gets 6His-tagged at the amino terminus (cursive). Downstream of the *Bam*HI site, the sequence coding for the TEV recognition site is introduced (*ENLYFQG* is highlighted) right before the mature toxin's sequence. Two stop codons (asterisks) are included at the end of the sequence coding for the mature toxin, upstream of the *Pst*I cloning site.

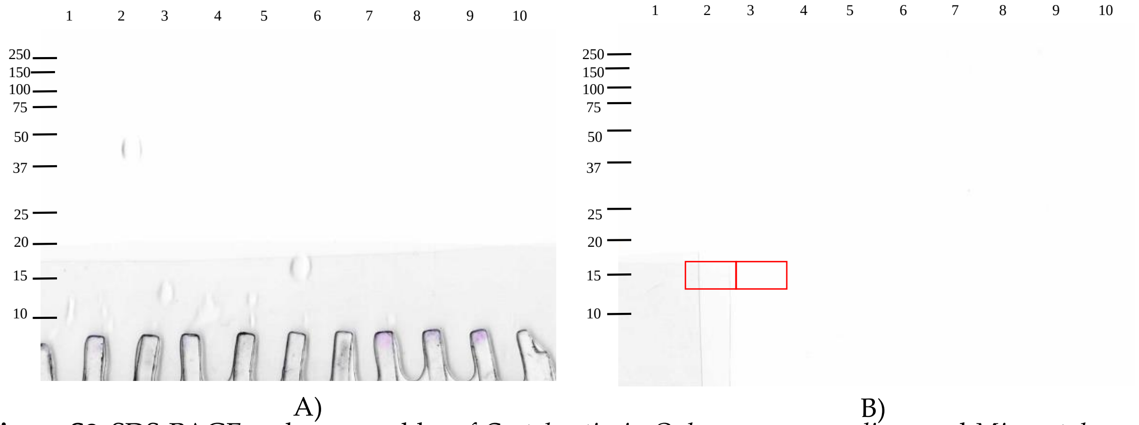

Figure S2. SDS-PAGE and western-blot of *Crotalus tigris, Ophryacus smaragdinus* and *Mixcuatylus melanurus* venoms. A) 15% SDS-PAGE gel; 1. MPM in kDa, 2. Purified crotoxin, 3. *Crotalus tigris* venom, 4. *Ophryacus smaragdinus* venom pool, 5. Individual HK909, 6. Individual HK910, 7. Individual HK610, 8. *Mixcuatylus melanurum* venom pool, 9. Individual HK625, 10. Individual HK626. B) Western-blot of the SDS-PAGE gel, 5 µg of native crotoxin and 10 µg of the venom pools and individual venom samples were loaded.

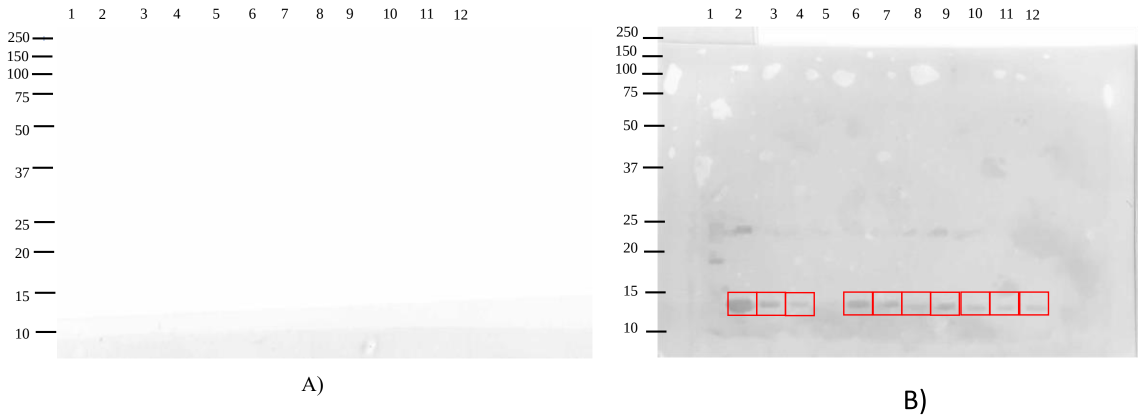

Figure S3. SDS-PAGE and western-blot of *Crotalus scutulatus scutulatus* venoms. A) 15% SDS-PAGE gel where 1. MPM in kDa, 2. Purified crotoxin, 3. *Crotalus scutulatus scutulatus* venom pool, 4. Individual HK343, 5. Individual HK538, 6. Individual HK504, 7. Individual HK345, 8 Individual HK344, 9. Individual HK503, 10. Individual HK375, 11. Individual HK347, 12. Individual HK346. B) Western-blot of the SDS-PAGE gel, 5 µg of native crotoxin and 10 µg of venom pool and individual venom samples were loaded.

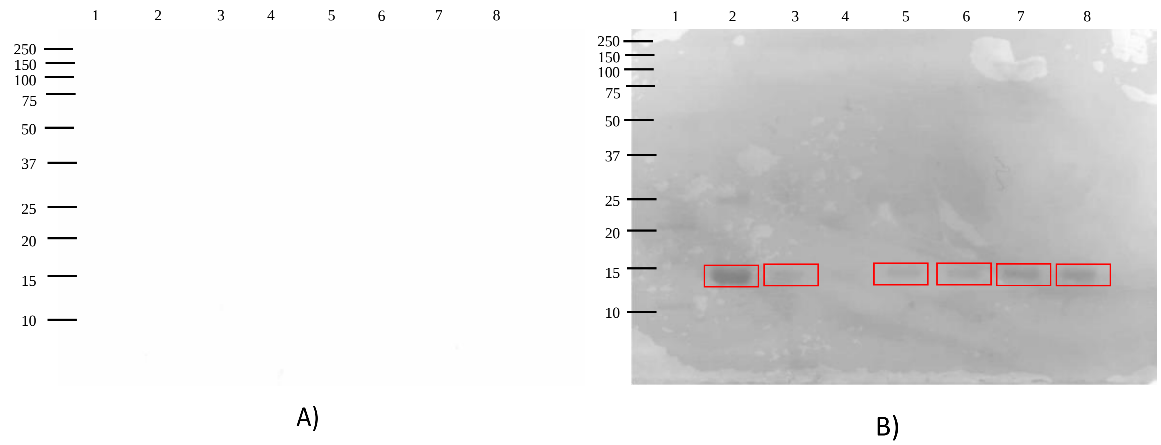

Figure S4. SDS-PAGE and western-blot of *Crotalus scutulatus salvini* venoms. A) 15% SDS-PAGE gel; 1. MPM in kDa, 2. Purified crotoxin, 3. *Crotalus scutulatus salvini* venom pool, 4. Individual HK873, 5. Individual HK872, 6. Individual HK871, 7. Individual HK870, 8 Individual HK858. B) Western-blot of the SDS-PAGE gel, 5 µg of native crotoxin and 10 µg of venom pool and individual venom samples were loaded.

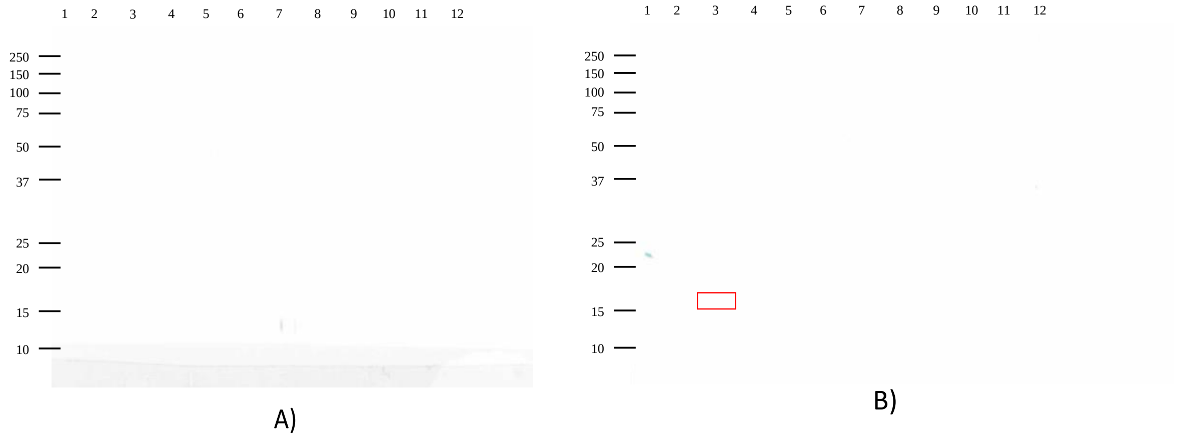

Figure S5. SDS-PAGE and western-blot of *Crotalus molossus nigrescens* venoms. A) 15% SDS-PAGE gel; 1. MPM in kDa, 2. Purified crotoxin, 3. *Crotalus molossus nigrescens* venom pool, 4. Individual HK839, 5. Individual HK837, 6. Individual HK396, 7. Individual HK320, 8. Individual HK354, 9. Individual HK342, 10. Individual HK353, 11. Individual HK352, 12. Individual HK351. B) Western-blot of the SDS-PAGE gel, 5 µg of native crotoxin and 10 µg of venom pool and individual venom samples were loaded.

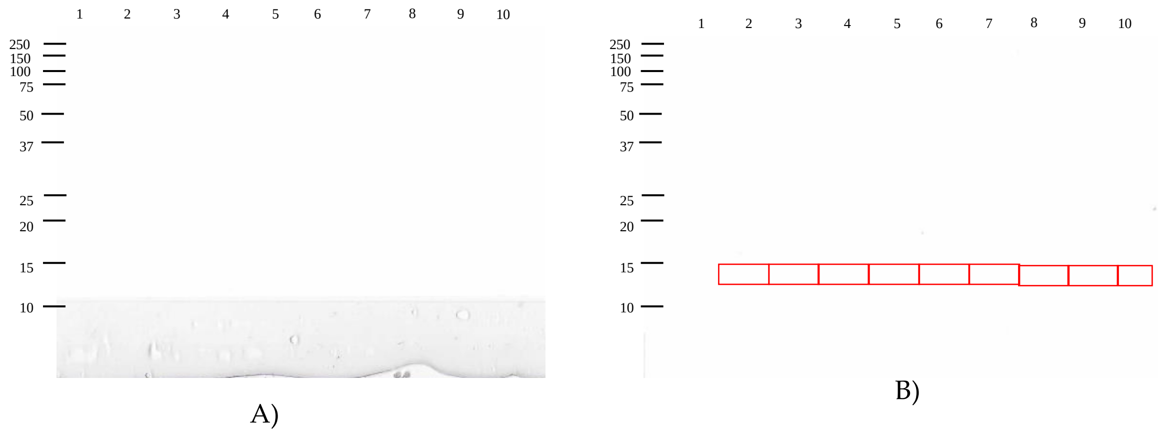

Figure S6. SDS-PAGE and western-blot of *Crotalus mictlantecuhtli* venoms. A) 15% SDS-PAGE gel; 1. MPM in kDa, 2. Purified crotoxin, 3. *Crotalus mictlantecuhtli* venom pool, 4. Individual HK900, 5. Individual HK878, 6. Individual HK899, 7. Individual HK977, 8. Individual HK869, 9. Individual HK876, 10. Individual HK857. B) Western-blot of the SDS-PAGE gel, 5 µg of native crotoxin and 10 µg of venom pool and individual venom samples were loaded.

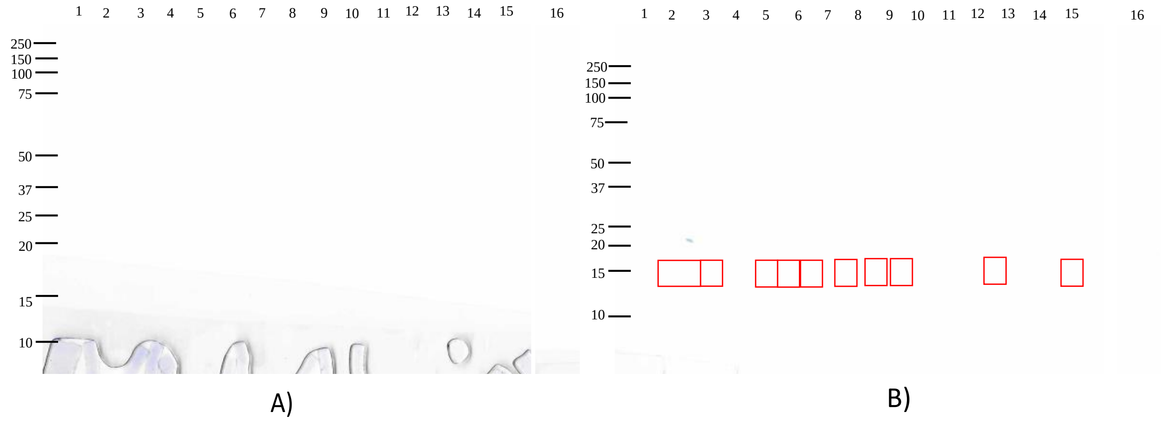

Figure S7. SDS-PAGE and western-blot of *Crotalus basiliscus* venoms. A) 15% SDS-PAGE gel; 1. MPM in kDa, 2. Purified crotoxin, 3. *Crotalus basiliscus* venom pool, 4. Individual HK455, 5. Individual HK454, 6. Individual HK453, 7. Individual HK442, 8. Individual HK451, 9. Individual HK450, 10. Individual HK447, 11. Individual HK441, 12. Individual HK440, 13. Individual HK381, 14. Individual HK439, 15. Individual HK300, 16. Individual HK326. B) Western-blot of the SDS-PAGE gel, 5 µg of native crotoxin and 10 µg of venom pool and individual venom samples were loaded.

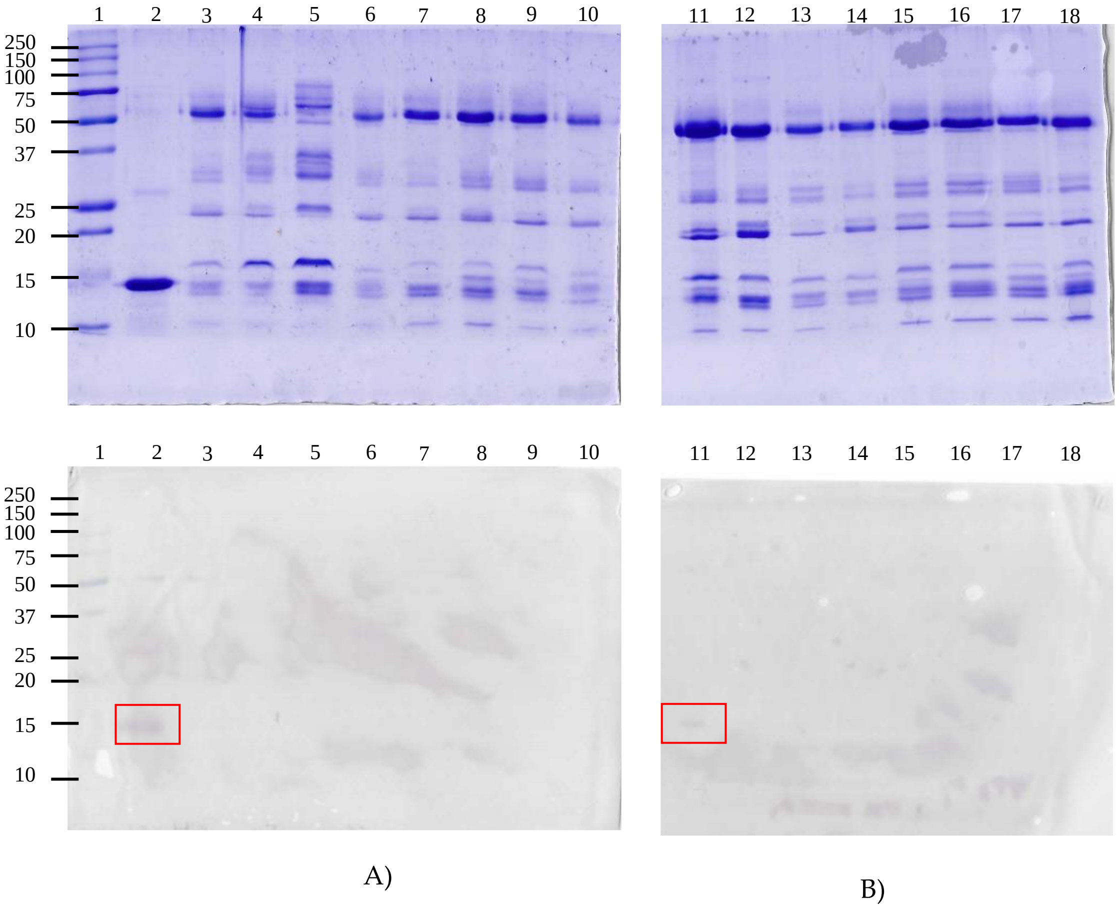

Figure S8. SDS-PAGE and western-blot of *Crotalus atrox* venoms. A) 15% SDS-PAGE gel; 1. MPM in kDa, 2. Purified crotoxin, 3. Pool of *Crotalus atrox* venoms, 4. Individual 854, 5. Individual Pool A, 6. Individual HK458, 7. Individual HK607, 8. Individual HK457, 9. Individual HK849, 10. Individual HK429, 11. Individual HK428, 12. Individual HK427, 13. Individual HK387, 14. Individual HK327, 15. Individual HK386, 16. Individual HK385, 17. Individual HK305, 18 Individual HK320. B) Western-blot of the SDS-PAGE gels, 5 μg of native crotoxin and 10 μg of venom pools and individual venom samples were loaded.

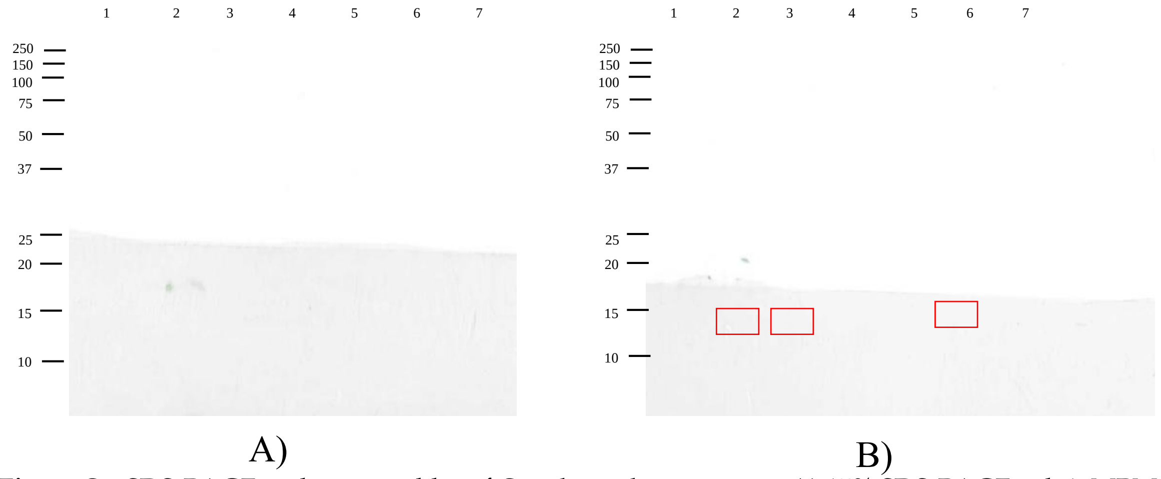

Figure S9. SDS-PAGE and western-blot of *Crotalus tzabcan* venoms. A) 15% SDS-PAGE gel; 1. MPM in kDa, 2. Purified crotoxin, 3. Crotalus *tzabcan* venom pool, 4. Individual HK751, 5. Individual HK879, 6. Individual HK897, 7. Individual HK898. B) Western-blot of the SDS-PAGE gel, 5 µg of native crotoxin and 10 µg of venom pool and individual venom samples were loaded.

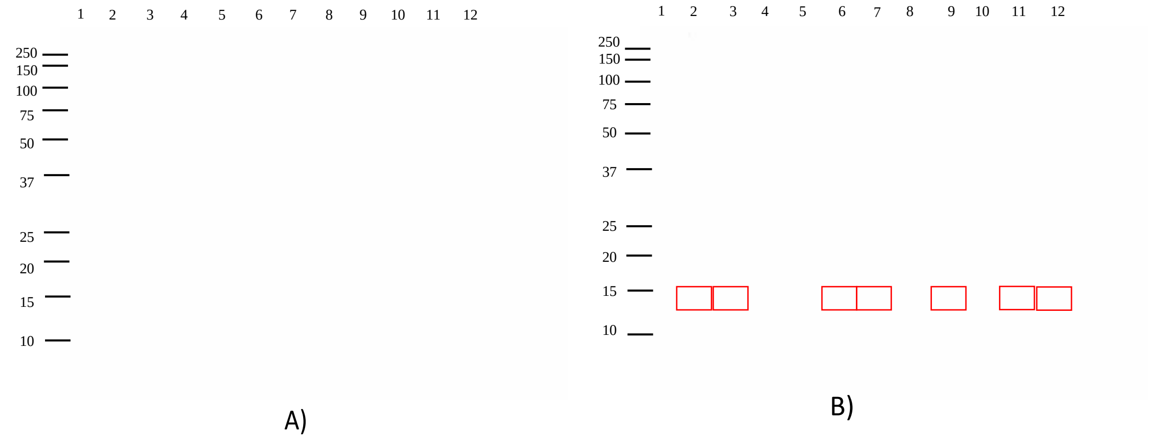

Figure S10. SDS-PAGE and western-blot of pools of crotalid venoms. A) 15% SDS-PAGE gel; 1. MPM in kDa, 2. Purified crotoxin, 3. *Crotalus tigris*, 4. *Ophryacus smaragdinus*, 5. *Mixcuatlus melanurum*, 6. *Crotalus scutulatus salvini*, 7. *Crotalus scutulatus scutulatus*, 8. *Crotalus molossus nigrescens*, 9. *Crotalus basiliscus*, 10. *Crotalus atrox*, 11. *Crotalus tzabcan*, 12. *Crotalus mictlantecuhtli*. B) Western-blot of the SDS-PAGE gel; 5 µg of native crotoxin and 15 µg of venom pool samples were loaded.
